# Huge peliosis hepatis mimicking cystic echinococcosis: A case report

**DOI:** 10.1097/MD.0000000000018141

**Published:** 2019-12-20

**Authors:** JinHeng Liu, YanTin Wang, SiNeng Yin, NengWen Ke, XuBao Liu

**Affiliations:** a Department of Pancreatic Surgery, West China Hospital, Sichuan University, Chengdu; b Department of Hepatobiliary-Pancreatic Surgery, Chengdu Second People's Hospital, Chengdu, Sichuan, China.

**Keywords:** cystic echinococcosis, diagnosis, immunohistochemistry, peliosis hepatis

## Abstract

**Rationale:**

Peliosis hepatis (PH), which is characterized by blood-filled cavities in the liver, is a rare disease. Its diagnosis depends on postoperative pathological examinations and immunohistochemistry.

**Patient concerns:**

A 44-year-old female complained of right-middle upper abdominal pain and distension for 1 month, with occasional vomiting and fever.

**Diagnosis:**

Because of the similar imaging features, the patient was initially misdiagnosed as cystic echinococcosis (CE). The immunoassay of echinococcosis was negative. Irregular hepatectomy was performed. Eventually, the patient was diagnosed with PH based on postoperative histopathology and immunohistochemistry.

**Interventions:**

The patient underwent hepatectomy. Then, the cystic lesion was collected for intraoperative pathological examination. Thus, the blood liquid was extracted from the cystic lesion. Pringle maneuver was administered to prevent bleeding, and then the whole cystic lesion was removed.

**Outcomes:**

She recovered smoothly and there was no relapse occurred during 6 months’ follow-up.

**Lessons:**

It is difficult to differentiate PH from CE and other hepatic diseases due to the lack of special imaging features. Pathological examinations and immunohistochemistry can provide a confirmed diagnosis of PH.

## 1 Introduction

Peliosis hepatis (PH), of which pathogenesis is unknown, is a rare benign lesion of the liver and characterized by the blood-filled cavities in liver.^[1]^ It often has no special imaging features, and its imaging features are often similar to those of other hepatic diseases.^[2]^ It is usually difficult to differentiate PH from other hepatic diseases at initial diagnostic stage.^[3]^ Herein, we reported a case of PH in a 44-year-old female who was initially misdiagnosed as cystic echinococcosis (CE) in another hospital because of the similar imaging features between the 2 diseases. She was finally diagnosed with PH according to the postoperative pathological examination and immunohistochemistry in our hospital.

## 2 Case presentation

**Figure 1:**
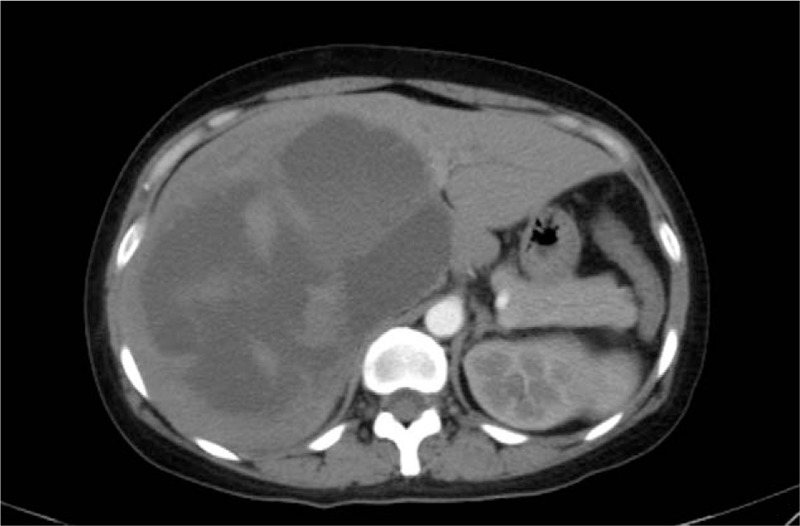
CT showed a huge cystic lesion sized 13.5 × 12 cm in the liver, and ascus and hemorrhage were observed in the lesion. CT = computed tomography.

**Figure 2:**
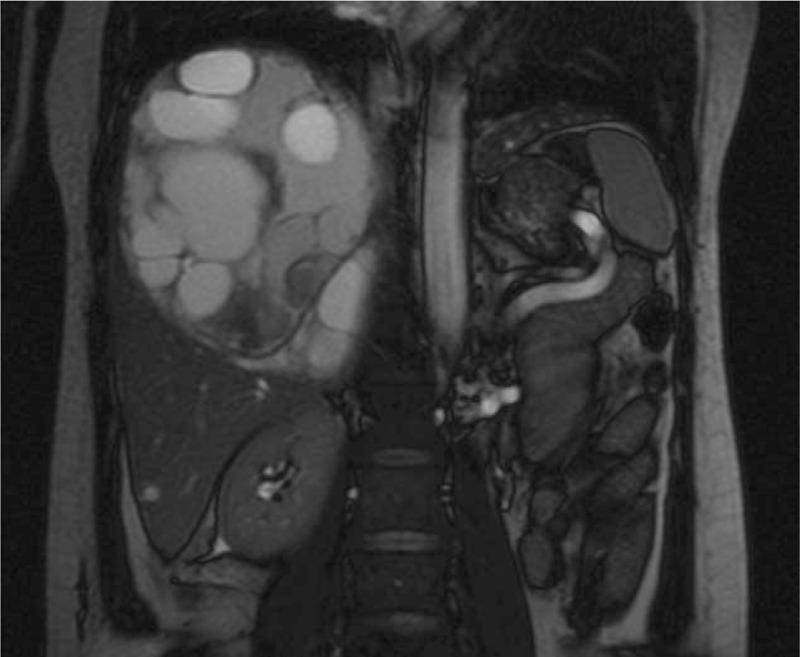
MRI showed a large cystic lesion containing smaller round shaped cystic lesions with hemorrhage and fluid. MRI = magnetic resonance imaging.

This study was approved by the Ethics Committee of Chengdu Second People's Hospital. A 44-year-old female was referred to our hospital because of complaint of right-middle upper abdominal pain and distension for 1 month, with occasional vomiting and fever. Enhanced computed tomography (CT) showed a huge cystic lesion sized 13.5 × 12 cm in the right lobe of the liver, and ascus and hemorrhage were observed in the lesion (Fig. 1). The periphery of the cystic lesion was enhanced in the arterial phase. Magnetic resonance imaging (MRI) also revealed a similar cystic lesion with surrounding enhancement (Fig. 2). Chest CT showed a small amount of fluid in the thorax. Routine laboratory tests showed red blood cell count, 2.76 × 10^9^/L (normal, 3.68–5.73 × 109/L); hemoglobin, 81 g/L (normal, 113–151 g/L); hematocrit, 26.1% (normal, 33.5%–45%); neutrophil granulocyte percentage, 73.2% (normal, 50%–70%); total bilirubin, 41.9 μmol/L (normal, 6–26 μmol/L); direct bilirubin, 17.6 μmol/L (normal, 0–11 μmol/L); indirect bilirubin, 24.3 μmol/L (normal, 3.4–17.1 μmol/L); prothrombin time, 12.9 seconds (normal, 9.3–12.4 seconds); plasm D-Dimer, 63.8 μg/mL (normal, 0–0.55); fibrinogen degradation product, 113.4 μg/mL (normal, 0–5). Besides, tumor markers such as α-fetoprotein, carcinoembryonic antigen and carbohydrate antigen 19-9 were in normal ranges. Serologic tests for complement C3/C4, kidney function, humoral immune antibody spectrum, hepatitis A/B/C and human immunodeficiency virus showed negative. Serum enzyme-linked immunosorbent assay for echinococcosis showed negative. Electrocardiography and gastroscopy showed no evident abnormalities. This patient had no history of living in echinococcosis endemic areas, eating raw beef and exposing to toxic agents or immunosuppressive drugs. According to the imaging features, she was initially diagnosed with CE, but the clinical manifestations should be taken into account in the clinical diagnosis as proposed by the radiologists.

**Figure 3:**
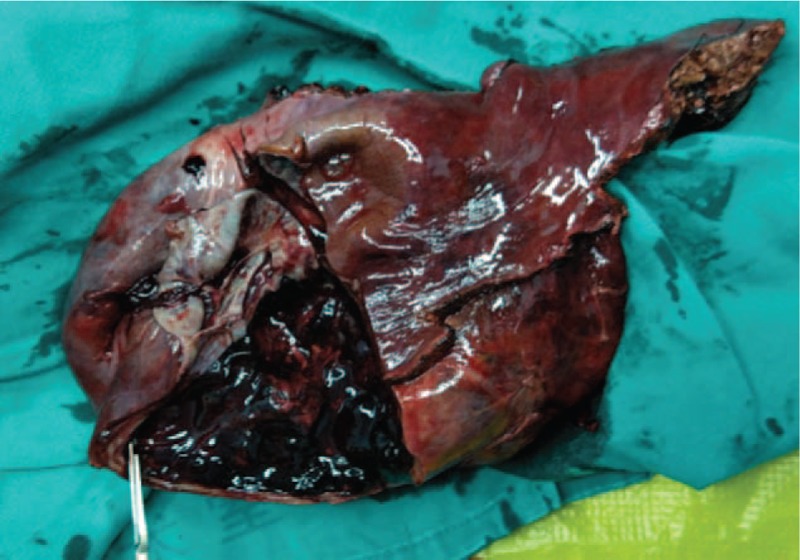
Hemorrhage in the lesion.

Then, a selective surgery was suggested for the removal of the hepatic lesion. Informed written consent was obtained from the patient for publication of this case report and accompanying images. Hepatectomy was performed. Following laparotomy, the hepatic surface was found to be smooth and the lesion almost ruptured (Fig. 3). Then, the cystic lesion was collected for the intraoperative pathological examination which revealed that it was benign. Thus, the blood liquid was extracted from the cystic lesion. Pringle maneuver was administered to prevent bleeding, and then the whole cystic lesion was removed. Autotransfusion was performed intraoperatively. The patient recovered smoothly after surgery and was in good health at 6-month postoperation.

**Figure 4:**
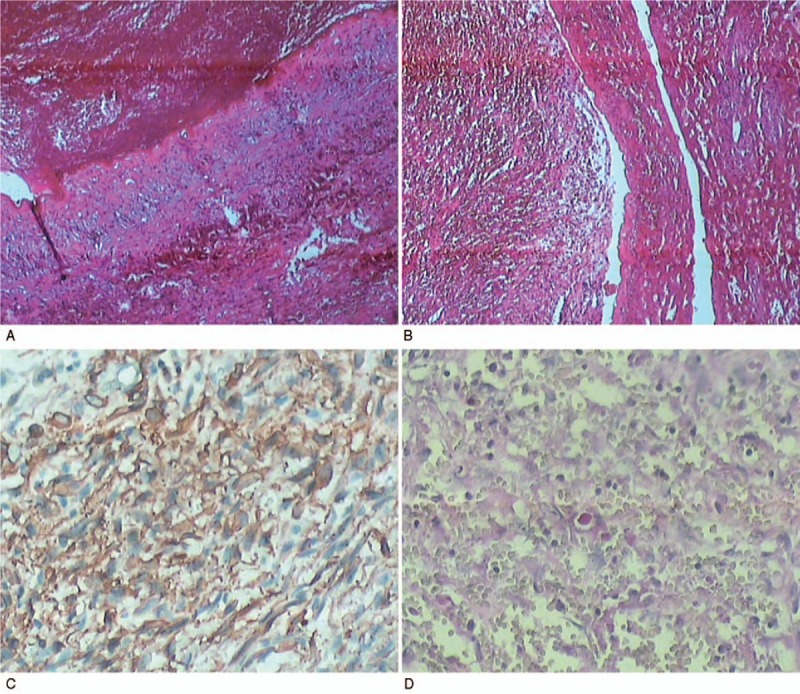
(A and B) Microscopically, blood-filled cysts and hemorrhagic necrosis were found to be close to the peliotic spaces without endothelial lining (A: 100×; B: 200×). (C and D) Immunohistochemistry showed the lesion was negative for CD31, CD34, CD117, DOG-1, PCK, EMA, HMB45, and F8 in the sinusoidal dilation area (C: 400×, D: 400×).

PH was diagnosed according to the postoperative pathological examination and immunohistochemistry. Microscopically, blood-filled cysts and hemorrhagic necrosis were observed, and the adjacent peliotic spaces had no endothelial lining, which are important pathological characteristics of PH. Immunohistochemistry showed the lesion was negative for CD31, CD34, CD117, DOG-1, PCK, EMA, HMB45, and F8 in the sinusoidal dilation area, but it was positive in the normal sinusoidal area (Fig. 4).

## 3 Discussion

PH is a rare benign lesion of the liver. Schoenlank named this disease for the first time in 1916.^[4]^ Recent studies proposed many mechanisms for the pathogenesis of PH.^[5]^ It was reported that the use of hormones and immunosuppressive agents such as methotrexate,^[6]^ tamoxifen,^[7]^ glucocorticoid,^[8,9]^ and azathioprim,^[10]^ are related to the pathogenesis of PH. Some diseases or pathological conditions, such as prostate cancer,^[10]^ immune deficiency after transplantation,^[11]^ hematological malignancies,^[12]^ and tuberculosis,^[13]^ may cause immune system dysfunction and reduce the autoimmunity, leading to the presence of PH. Infection due to immunodeficiency may also be a pathogenic factor of PH, such as acquired immunodeficiency syndrome and its secondary infection.^[13]^ And Bartonella infection observed in PH dogs is also proposed as a possible cause of this disease.^[14]^ One or more of these factors may cause hepatocyte necrosis which then leads to the formation of cysts^[15]^; in addition, the dysfunctional endothelial cells in the hepatic sinus may cause angiectasis and hyperemia, leading to the formation of mass blood-filled cysts in the hepatic parenchyma.^[16]^

Although the patient was diagnosed as CE by radiologists, she had no history of living in epidemic areas, eating raw beef, and exposing to toxic agents and immunosuppressants. Thus, the specific mechanism for the pathogenesis of PH was unknown. In the diagnosis of CE, CT, and MRI play a significant role and they can display the cystic size, location, and relations to adjacent organs. In a previous report of CE, CT showed a large cystic lesion containing smaller round shaped cystic lesions with a lower density inner substance than in the large cystic lesion.^[17]^ Enhanced imaging examination of CE shows different degrees of eggshell or punctate calcification in both large and its inner cyst wall. MRI shows the large cystic lesion had stronger signals on T1WI (T1 weighted imaging) and weaker signals on T2WI (T2 weighted imaging) than those of its inner cystic lesions, and the cyst wall had consistently low signals.^[18]^ Nonetheless, PH usually shows centrifugal enhancement in venous phase and centripetal enhancement in the arterial phase on CT. Because of intracavity hemorrhage, PH shows the lesion with different signals in MRI images.^[19]^ Previous literature had reported that PH had long T1WI and short T2WI signal in the hemorrhagic cystic lesion in the subacute phase, and long T1WI and T2WI signal in chronic hemorrhage phase.^[20]^ The remote and recurrent hemorrhage may display fluid interface.^[21]^ We reviewed all the imaging features of this patient. MRI of this patient showed a lesion in the right liver, sized approximately 13.5 × 12 cm with hemorrhage and fluid in it, and long T1WI and T2WI signals were observed in the lesion. PH and echinococcosis have a similar specific imaging feature of multiple inner small cysts in the large cyst. Therefore, the PH mimicking CE has not been reported in previous literature.

In this case, the patient had no previous medical history. Though the serologic tests for echinococcosis showed negative, the imaging features of PH mimicking CE made diagnosis difficult. Therefore, pathological examination and immunohistochemistry could definitely diagnose PH. In addition, PH should be differentiated from other hepatic diseases such as hepatic adenoma, hemangioma, focal nodular hyperplasia, and hepatic abscess.^[22]^

In this patient, the moderate anemia might be caused by hemorrhage in the lesion. Surgery is the most effective treatment for the prevention of massive hemorrhage caused by lesion rupture,^[23]^ and hepatic failure.^[24]^ The preoperative liver function (Child-Pugh grade A) of this patient was evaluated after surgery, and results showed normal. Then, a precise hepatectomy was performed to ensure that the liver tissues could be reserved as more as possible to prevent postoperative hepatic failure. Echinococcosis requires surgical treatment and long-term medication to avoid recurrence. Compared to echinococcosis, patients in previous literature reports were followed up with no recurrence after surgical resection and could live for long term survival. And the patient was no relapse occurred during 6 months’ follow-up.

## Author contributions

**Conceptualization:** JinHeng Liu, YanTin Wang, Xubao Liu.

**Data curation:** JinHeng Liu, YanTin Wang, SiNeng Yin, NengWen Ke.

**Formal analysis:** JinHeng Liu.

**Funding acquisition:** Xubao Liu.

**Investigation:** JinHeng Liu, YanTin Wang.

**Methodology:** JinHeng Liu, SiNeng Yin.

**Resources:** SiNeng Yin, NengWen Ke.

**Visualization:** Xubao Liu.

**Writing – original draft:** JinHeng Liu, YanTin Wang.

**Writing – review and editing:** Xubao Liu.
